# *O*-Benzoyl­naltrexone

**DOI:** 10.1107/S1600536813016036

**Published:** 2013-06-15

**Authors:** Rui Yang, Guo-Hai Wang, Xia-Li Liu, Dong Wang, Xiang Li

**Affiliations:** a Department of Pharmacology, Technical Center, Jiangsu Nhwa Pharma Corporation, Zhongshan Road No. 289, Xuzhou 221009, People’s Republic of China; b Key Laboratory of Coal Processing and Efficient Utilization, (Ministry of Education), China University of Mining & Technology, Xuzhou 221116, Jiangsu, People’s Republic of China

## Abstract

In the title compound, C_27_H_27_NO_5_ (systematic name: 17-cyclopropylmethyl-14-hydroxy-6-oxo-4,5-epoxymorphin­an-6-yl benzoate), which is the benzoate ester of the opioid receptor antagonist naltrexone, the dihedral angle between the two phenyl rings is 77.1 (1)°. In the crystal, a weak aromatic C—H⋯O_carbox­yl_ hydrogen bond involving the benzoate groups of adjacent mol­ecules gives rise to a chain extending along the *a*-axis direction. The known absolute configuration for the mol­ecule was inferred from a previous naltrexone structure.

## Related literature

For chemical properties of naltrexone, see: Fernando *et al.* (2008 ▶); Beznischenko *et al.* (2007 ▶). For related structures, see: Ledain *et al.* (1992 ▶); Li *et al.* (2012 ▶).
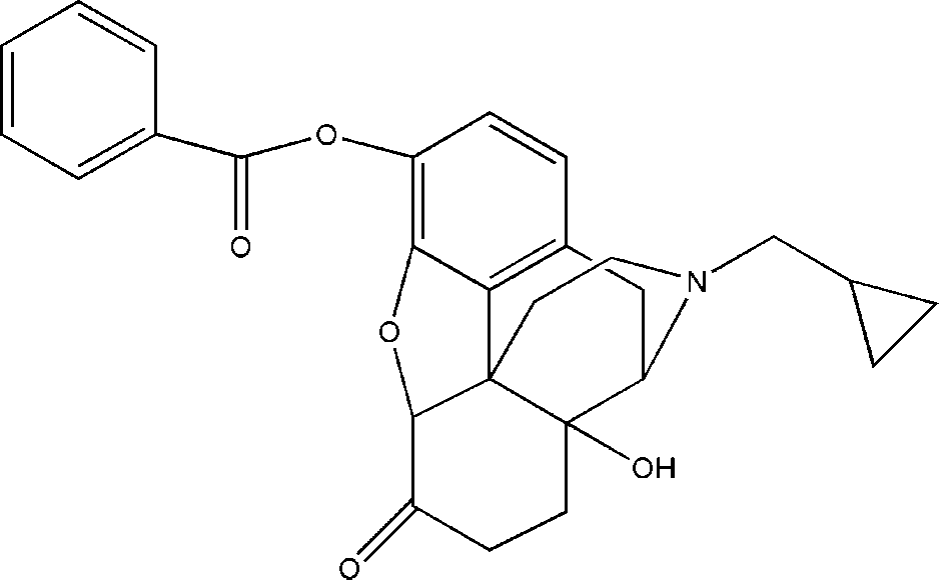


## Experimental

### 

#### Crystal data

C_27_H_27_NO_5_
*M* *_r_* = 445.50
Monoclinic, 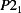

*a* = 7.8890 (16) Å
*b* = 8.6620 (17) Å
*c* = 16.629 (3) Å
β = 102.24 (3)°
*V* = 1110.5 (4) Å^3^
*Z* = 2
Mo *K*α radiation
μ = 0.09 mm^−1^
*T* = 293 K
0.30 × 0.20 × 0.10 mm

#### Data collection

Enraf–Nonius CAD-4 diffractometer
Absorption correction: ψ scan (*CAD-4 EXPRESS*; Enraf–Nonius, 1994 ▶) *T*_min_ = 0.973, *T*_max_ = 0.991
4421 measured reflections
4083 independent reflections
2611 reflections with *I* > 2σ(*I*)
*R*_int_ = 0.030
3 standard reflections every 200 reflections intensity decay: 1%

#### Refinement

*R*[*F* ^2^ > 2σ(*F* ^2^)] = 0.067
*wR*(*F* ^2^) = 0.180
*S* = 1.00
4083 reflections
301 parameters
1 restraint
H atoms treated by a mixture of independent and constrained refinement
Δρ_max_ = 0.23 e Å^−3^
Δρ_min_ = −0.23 e Å^−3^
Absolute structure: Flack (1983 ▶), 1886 Friedel pairs
Flack parameter: 0.04 (2)

### 

Data collection: *CAD-4 EXPRESS* (Enraf–Nonius, 1994 ▶); cell refinement: *CAD-4 EXPRESS*; data reduction: *XCAD4* (Harms & Wocadlo, 1995 ▶); program(s) used to solve structure: *SHELXS97* (Sheldrick, 2008 ▶); program(s) used to refine structure: *SHELXL97* (Sheldrick, 2008 ▶); molecular graphics: *DIAMOND* (Brandenburg & Putz, 2005 ▶) and *ORTEPIII* (Burnett & Johnson, 1996 ▶); software used to prepare material for publication: *SHELXL97*.

## Supplementary Material

Crystal structure: contains datablock(s) I, global. DOI: 10.1107/S1600536813016036/zs2258sup1.cif

Structure factors: contains datablock(s) I. DOI: 10.1107/S1600536813016036/zs2258Isup2.hkl

Additional supplementary materials:  crystallographic information; 3D view; checkCIF report

## Figures and Tables

**Table 1:** Hydrogen-bond geometry (Å, °)

*D*—H⋯*A*	*D*—H	H⋯*A*	*D*⋯*A*	*D*—H⋯*A*
C24—H24*A*⋯O5^i^	0.93	2.54	3.453 (7)	168

Symmetry code: (i) 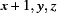.

**Fig. 1.**
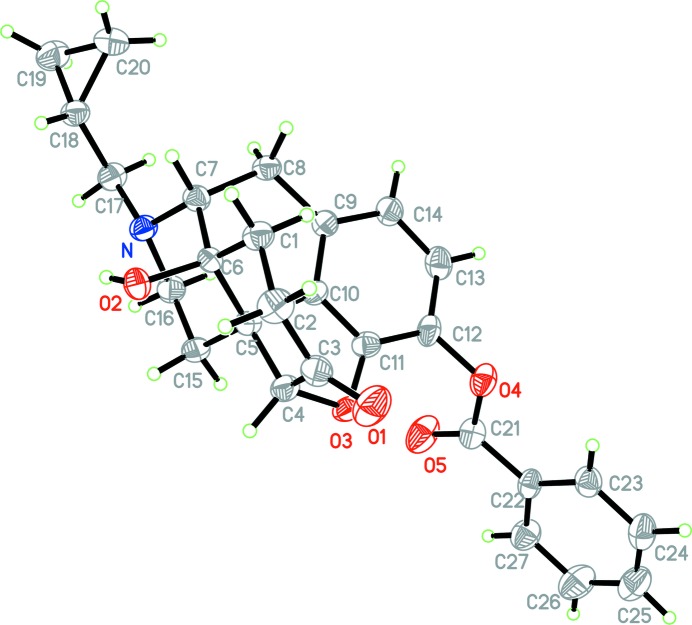
Molecular configuration and atom numbering scheme for the title compound, with displacement parameters drawn at the 40% probability level.

## supplementary crystallographic information

### Comment

The title compound C_27_H_27_NO_5_ is the benzoate ester of the opioid receptor antagonist naltrexone and is important as an intermediate for the preparation *N*-methylnaltrexone bromide. It is used for the treatment of a number of diseases which are related to abnormal release of endogenous opium (Beznischenko *et al.*, 2007). The structures of a number of derivatives of naltrexone are known, e.g. naltrexone hydrochloride dihydrate (Ledain *et al.*, 1992) and methylnaltrexone hydrobromide methanol monosolvate (Li *et al.*, 2012). In the title compound, the known absolute configuration for the molecule was inferred from a previous naltrexone structure (Li *et al.*, 2012).

In the crystal, a weak aromatic C—H···O_carboxyl_ hydrogen bond involving the benzoate moieties of adjacent molecules (Table 1) gives a one-dimensional chain extending along the *a* axial direction. Present also in the structure is an intramolecular O2—H···N interaction.

### Experimental

The title compound was prepared from 2.0 g (5.9 mmol) of naltrexone ([5α]-17-(cyclopropylmethyl-4,5-epoxy-3,14-dihydroxymorphinan-6-one)), 0.60 g of triethylamine and 16 ml of dichloromethane which were were successively introduced into a 100 ml reactor equipped with a condenser and a mechanical stirrer. After the solid had dissolved, benzoyl chloride (0.85 g, 6 mmol) was added over a 10 minute period at 20 °C and the reaction medium was refluxed for 2 h. The dichloromethane was removed under vacuum and the solid was recrystallized from ethanol, giving the pure title compound.

### Refinement

All H atoms attached to C atoms and N atom were fixed geometrically and treated as riding with C—H = 0.96 Å (methyl) or 0.97 Å (methylene) and N—H = 0.86 Å with *U*_iso_(H) = 1.2*U*_eq_(C or N) or *U*_iso_(H) = 1.5*U*_eq_(methyl). The hydroxy H-atom was located in a difference Fourier and included in the subsequent refinement using restraints [O—H = 0.85 (1) Å, with *U*_iso_(H) = 1.5*U*_eq_(O)]. The absolute structure [(C4*R*,C5*S*,C6*S*,C7*R*) for the current trivial atom numbering scheme] was inferred from a previous structure determination (Li *et al.*, 2012) [Flack structure parameter (Flack, 1983) for the present compound = 0.04 (2) for 1886 Friedel pairs].

### Crystal data

**Table:**

C_27_H_27_NO_5_	*F*(000) = 472
*M**_r_* = 445.50	*D*_x_ = 1.332 Mg m^−^^3^
Monoclinic, *P*2_1_	Mo *K*α radiation, λ = 0.71073 Å
Hall symbol: P 2yb	Cell parameters from 25 reflections
*a* = 7.8890 (16) Å	θ = 9–12°
*b* = 8.6620 (17) Å	µ = 0.09 mm^−^^1^
*c* = 16.629 (3) Å	*T* = 293 K
β = 102.24 (3)°	Block, colorless
*V* = 1110.5 (4) Å^3^	0.30 × 0.20 × 0.10 mm
*Z* = 2	

### Data collection

**Table:**

Enraf–Nonius CAD-4 diffractometer	2611 reflections with *I* > 2σ(*I*)
Radiation source: fine-focus sealed tube	*R*_int_ = 0.030
Graphite monochromator	θ_max_ = 25.4°, θ_min_ = 1.3°
ω/2θ scans	*h* = 0→9
Absorption correction: ψ scan (*CAD-4 EXPRESS*; Enraf–Nonius, 1994)	*k* = −10→10
*T*_min_ = 0.973, *T*_max_ = 0.991	*l* = −20→19
4421 measured reflections	3 standard reflections every 200 reflections
4083 independent reflections	intensity decay: 1%

### Refinement

**Table:**

Refinement on *F*^2^	Secondary atom site location: difference Fourier map
Least-squares matrix: full	Hydrogen site location: inferred from neighbouring sites
*R*[*F*^2^ > 2σ(*F*^2^)] = 0.067	H atoms treated by a mixture of independent and constrained refinement
*wR*(*F*^2^) = 0.180	*w* = 1/[σ^2^(*F*_o_^2^) + (0.095*P*)^2^] where *P* = (*F*_o_^2^ + 2*F*_c_^2^)/3
*S* = 1.00	(Δ/σ)_max_ < 0.001
4083 reflections	Δρ_max_ = 0.23 e Å^−^^3^
301 parameters	Δρ_min_ = −0.23 e Å^−^^3^
1 restraint	Absolute structure: Flack (1983), 1886 Friedel pairs
Primary atom site location: structure-invariant direct methods	Flack parameter: 0.04 (2)

### Special details

**Table:**

Geometry. All e.s.d.'s (except the e.s.d. in the dihedral angle between two l.s. planes) are estimated using the full covariance matrix. The cell e.s.d.'s are taken into account individually in the estimation of e.s.d.'s in distances, angles and torsion angles; correlations between e.s.d.'s in cell parameters are only used when they are defined by crystal symmetry. An approximate (isotropic) treatment of cell e.s.d.'s is used for estimating e.s.d.'s involving l.s. planes.
Refinement. Refinement of *F*^2^ against ALL reflections. The weighted *R*-factor *wR* and goodness of fit *S* are based on *F*^2^, conventional *R*-factors *R* are based on *F*, with *F* set to zero for negative *F*^2^. The threshold expression of *F*^2^ > σ(*F*^2^) is used only for calculating *R*-factors(gt) *etc*. and is not relevant to the choice of reflections for refinement. *R*-factors based on *F*^2^ are statistically about twice as large as those based on *F*, and *R*- factors based on ALL data will be even larger.

### Fractional atomic coordinates and isotropic or equivalent isotropic displacement parameters (Å^2^)

**Table:**

	*x*	*y*	*z*	*U*_iso_*/*U*_eq_	
N	0.2654 (5)	0.1880 (4)	0.6679 (2)	0.0435 (9)	
O2	0.3797 (4)	0.2516 (4)	0.53015 (19)	0.0510 (9)	
H2A	0.284 (7)	0.255 (8)	0.544 (4)	0.076*	
O1	0.9546 (5)	0.4322 (5)	0.5720 (2)	0.0767 (12)	
O3	0.8160 (4)	0.5154 (3)	0.7024 (2)	0.0534 (9)	
O4	1.0794 (4)	0.4753 (4)	0.8555 (2)	0.0543 (9)	
O5	0.9070 (5)	0.6447 (5)	0.8988 (3)	0.0809 (13)	
C1	0.6515 (6)	0.1322 (5)	0.5711 (3)	0.0460 (12)	
H1A	0.6060	0.0460	0.5358	0.055*	
H1B	0.7364	0.0922	0.6173	0.055*	
C2	0.7392 (7)	0.2459 (6)	0.5232 (3)	0.0565 (13)	
H2B	0.8310	0.1930	0.5036	0.068*	
H2C	0.6550	0.2812	0.4754	0.068*	
C3	0.8144 (7)	0.3834 (6)	0.5732 (3)	0.0531 (13)	
C4	0.7009 (6)	0.4580 (6)	0.6267 (3)	0.0513 (12)	
H4A	0.6374	0.5451	0.5969	0.062*	
C5	0.5727 (6)	0.3498 (5)	0.6553 (3)	0.0432 (11)	
C6	0.5069 (5)	0.2075 (5)	0.6020 (3)	0.0376 (10)	
C7	0.4182 (6)	0.1033 (5)	0.6559 (3)	0.0419 (11)	
H7A	0.3753	0.0126	0.6226	0.050*	
C8	0.5416 (6)	0.0425 (5)	0.7345 (3)	0.0444 (11)	
H8A	0.4735	0.0151	0.7746	0.053*	
H8B	0.5975	−0.0507	0.7208	0.053*	
C9	0.6798 (6)	0.1565 (5)	0.7733 (3)	0.0405 (10)	
C10	0.6907 (5)	0.2929 (5)	0.7329 (3)	0.0390 (10)	
C11	0.8196 (6)	0.3975 (5)	0.7578 (3)	0.0437 (11)	
C12	0.9405 (6)	0.3749 (6)	0.8295 (3)	0.0485 (12)	
C13	0.9307 (6)	0.2405 (6)	0.8727 (3)	0.0554 (13)	
H13A	1.0107	0.2228	0.9215	0.067*	
C14	0.8052 (6)	0.1324 (6)	0.8448 (3)	0.0506 (12)	
H14A	0.8038	0.0413	0.8743	0.061*	
C15	0.4194 (6)	0.4364 (5)	0.6763 (3)	0.0497 (12)	
H15A	0.4620	0.5205	0.7137	0.060*	
H15B	0.3495	0.4804	0.6265	0.060*	
C16	0.3093 (6)	0.3310 (5)	0.7155 (3)	0.0495 (12)	
H16A	0.3710	0.3053	0.7707	0.059*	
H16B	0.2032	0.3841	0.7197	0.059*	
C17	0.1400 (6)	0.0976 (5)	0.7005 (3)	0.0492 (12)	
H17A	0.0418	0.1631	0.7035	0.059*	
H17B	0.1929	0.0660	0.7561	0.059*	
C18	0.0748 (6)	−0.0432 (5)	0.6513 (3)	0.0465 (11)	
H18A	0.0414	−0.0291	0.5915	0.056*	
C19	−0.0330 (7)	−0.1532 (6)	0.6878 (4)	0.0650 (15)	
H19A	−0.0513	−0.1308	0.7425	0.078*	
H19B	−0.1307	−0.2015	0.6512	0.078*	
C20	0.1424 (7)	−0.1989 (6)	0.6801 (4)	0.0617 (14)	
H20A	0.1524	−0.2752	0.6386	0.074*	
H20B	0.2318	−0.2044	0.7300	0.074*	
C21	1.0464 (7)	0.6142 (6)	0.8869 (3)	0.0485 (12)	
C22	1.2002 (6)	0.7137 (6)	0.9061 (3)	0.0442 (11)	
C23	1.3556 (6)	0.6722 (6)	0.8883 (3)	0.0517 (12)	
H23A	1.3666	0.5779	0.8632	0.062*	
C24	1.4963 (7)	0.7707 (7)	0.9079 (3)	0.0631 (15)	
H24A	1.6025	0.7417	0.8968	0.076*	
C25	1.4796 (8)	0.9124 (8)	0.9439 (4)	0.0750 (17)	
H25A	1.5730	0.9803	0.9555	0.090*	
C26	1.3254 (8)	0.9509 (8)	0.9622 (4)	0.0793 (18)	
H26A	1.3145	1.0444	0.9881	0.095*	
C27	1.1857 (7)	0.8539 (6)	0.9430 (3)	0.0604 (14)	
H27B	1.0802	0.8829	0.9549	0.073*	

### Atomic displacement parameters (Å^2^)

**Table:**

	*U*^11^	*U*^22^	*U*^33^	*U*^12^	*U*^13^	*U*^23^
N	0.043 (2)	0.037 (2)	0.050 (2)	−0.0027 (17)	0.0110 (18)	−0.0051 (18)
O2	0.0522 (19)	0.054 (2)	0.0395 (17)	−0.0002 (18)	−0.0069 (16)	0.0060 (15)
O1	0.066 (2)	0.093 (3)	0.073 (3)	−0.024 (2)	0.020 (2)	0.003 (2)
O3	0.061 (2)	0.0400 (18)	0.0533 (19)	−0.0179 (16)	−0.0021 (16)	0.0009 (16)
O4	0.0430 (19)	0.063 (2)	0.055 (2)	−0.0057 (17)	0.0040 (16)	−0.0182 (17)
O5	0.057 (2)	0.081 (3)	0.111 (3)	−0.012 (2)	0.033 (2)	−0.040 (3)
C1	0.055 (3)	0.044 (3)	0.037 (2)	−0.007 (2)	0.007 (2)	−0.002 (2)
C2	0.066 (3)	0.064 (3)	0.044 (3)	−0.006 (3)	0.020 (2)	0.003 (3)
C3	0.055 (3)	0.058 (3)	0.044 (3)	−0.004 (3)	0.008 (2)	0.021 (2)
C4	0.054 (3)	0.045 (3)	0.049 (3)	−0.012 (2)	−0.002 (2)	0.010 (2)
C5	0.047 (3)	0.033 (2)	0.046 (3)	−0.003 (2)	0.003 (2)	0.003 (2)
C6	0.038 (2)	0.036 (2)	0.037 (2)	0.0006 (19)	0.0028 (19)	0.0057 (19)
C7	0.045 (3)	0.039 (2)	0.041 (2)	0.002 (2)	0.008 (2)	−0.001 (2)
C8	0.050 (3)	0.037 (2)	0.047 (3)	−0.004 (2)	0.011 (2)	0.004 (2)
C9	0.043 (3)	0.043 (3)	0.034 (2)	−0.002 (2)	0.007 (2)	0.002 (2)
C10	0.042 (2)	0.036 (2)	0.038 (2)	−0.002 (2)	0.005 (2)	−0.006 (2)
C11	0.052 (3)	0.038 (3)	0.041 (3)	−0.003 (2)	0.010 (2)	−0.007 (2)
C12	0.037 (3)	0.059 (3)	0.044 (3)	−0.004 (2)	−0.002 (2)	−0.012 (2)
C13	0.052 (3)	0.071 (4)	0.038 (3)	0.002 (3)	−0.001 (2)	0.005 (3)
C14	0.050 (3)	0.057 (3)	0.042 (3)	−0.004 (3)	0.004 (2)	0.012 (2)
C15	0.055 (3)	0.033 (2)	0.057 (3)	−0.003 (2)	0.002 (2)	−0.007 (2)
C16	0.051 (3)	0.042 (3)	0.055 (3)	0.002 (2)	0.009 (2)	−0.011 (2)
C17	0.046 (3)	0.048 (3)	0.057 (3)	−0.001 (2)	0.017 (2)	−0.005 (2)
C18	0.053 (3)	0.041 (3)	0.045 (3)	−0.005 (2)	0.010 (2)	0.002 (2)
C19	0.066 (4)	0.057 (3)	0.074 (4)	−0.011 (3)	0.018 (3)	0.001 (3)
C20	0.077 (4)	0.045 (3)	0.065 (3)	0.001 (3)	0.019 (3)	0.005 (2)
C21	0.044 (3)	0.059 (3)	0.043 (3)	−0.001 (2)	0.011 (2)	−0.008 (2)
C22	0.037 (2)	0.061 (3)	0.034 (2)	−0.005 (2)	0.0051 (19)	−0.001 (2)
C23	0.048 (3)	0.061 (3)	0.043 (3)	0.001 (3)	0.003 (2)	0.003 (2)
C24	0.046 (3)	0.090 (4)	0.054 (3)	−0.001 (3)	0.011 (2)	0.006 (3)
C25	0.068 (4)	0.085 (5)	0.068 (4)	−0.026 (3)	0.006 (3)	−0.003 (3)
C26	0.087 (4)	0.072 (4)	0.079 (4)	−0.023 (4)	0.019 (4)	−0.029 (3)
C27	0.059 (3)	0.059 (3)	0.064 (3)	−0.007 (3)	0.015 (3)	−0.014 (3)

### Geometric parameters (Å, º)

**Table:**

N—C17	1.453 (6)	C11—C12	1.374 (6)
N—C7	1.460 (6)	C12—C13	1.378 (7)
N—C16	1.471 (5)	C13—C14	1.370 (7)
O2—C6	1.440 (5)	C13—H13A	0.9300
O2—H2A	0.84 (5)	C14—H14A	0.9300
O1—C3	1.188 (6)	C15—C16	1.501 (7)
O3—C11	1.372 (5)	C15—H15A	0.9700
O3—C4	1.473 (6)	C15—H15B	0.9700
O4—C21	1.358 (6)	C16—H16A	0.9700
O4—C12	1.394 (5)	C16—H16B	0.9700
O5—C21	1.187 (6)	C17—C18	1.498 (7)
C1—C6	1.496 (6)	C17—H17A	0.9700
C1—C2	1.522 (6)	C17—H17B	0.9700
C1—H1A	0.9700	C18—C19	1.490 (7)
C1—H1B	0.9700	C18—C20	1.491 (7)
C2—C3	1.501 (7)	C18—H18A	0.9800
C2—H2B	0.9700	C19—C20	1.471 (7)
C2—H2C	0.9700	C19—H19A	0.9700
C3—C4	1.534 (7)	C19—H19B	0.9700
C4—C5	1.527 (6)	C20—H20A	0.9700
C4—H4A	0.9800	C20—H20B	0.9700
C5—C10	1.505 (6)	C21—C22	1.467 (6)
C5—C15	1.526 (6)	C22—C23	1.369 (6)
C5—C6	1.542 (6)	C22—C27	1.376 (7)
C6—C7	1.541 (6)	C23—C24	1.383 (7)
C7—C8	1.547 (6)	C23—H23A	0.9300
C7—H7A	0.9800	C24—C25	1.384 (9)
C8—C9	1.510 (6)	C24—H24A	0.9300
C8—H8A	0.9700	C25—C26	1.357 (8)
C8—H8B	0.9700	C25—H25A	0.9300
C9—C10	1.371 (6)	C26—C27	1.369 (8)
C9—C14	1.393 (6)	C26—H26A	0.9300
C10—C11	1.359 (6)	C27—H27B	0.9300
			
C17—N—C7	115.3 (4)	C14—C13—C12	121.2 (4)
C17—N—C16	110.7 (3)	C14—C13—H13A	119.4
C7—N—C16	112.9 (3)	C12—C13—H13A	119.4
C6—O2—H2A	107 (4)	C13—C14—C9	121.2 (5)
C11—O3—C4	104.1 (3)	C13—C14—H14A	119.4
C21—O4—C12	118.0 (4)	C9—C14—H14A	119.4
C6—C1—C2	111.2 (4)	C16—C15—C5	111.1 (4)
C6—C1—H1A	109.4	C16—C15—H15A	109.4
C2—C1—H1A	109.4	C5—C15—H15A	109.4
C6—C1—H1B	109.4	C16—C15—H15B	109.4
C2—C1—H1B	109.4	C5—C15—H15B	109.4
H1A—C1—H1B	108.0	H15A—C15—H15B	108.0
C3—C2—C1	113.2 (4)	N—C16—C15	111.7 (4)
C3—C2—H2B	108.9	N—C16—H16A	109.3
C1—C2—H2B	108.9	C15—C16—H16A	109.3
C3—C2—H2C	108.9	N—C16—H16B	109.3
C1—C2—H2C	108.9	C15—C16—H16B	109.3
H2B—C2—H2C	107.8	H16A—C16—H16B	107.9
O1—C3—C2	122.3 (5)	N—C17—C18	114.7 (4)
O1—C3—C4	121.3 (5)	N—C17—H17A	108.6
C2—C3—C4	116.4 (4)	C18—C17—H17A	108.6
O3—C4—C5	105.7 (4)	N—C17—H17B	108.6
O3—C4—C3	107.9 (4)	C18—C17—H17B	108.6
C5—C4—C3	115.4 (4)	H17A—C17—H17B	107.6
O3—C4—H4A	109.2	C19—C18—C20	59.1 (3)
C5—C4—H4A	109.2	C19—C18—C17	117.3 (4)
C3—C4—H4A	109.2	C20—C18—C17	120.2 (4)
C10—C5—C15	109.9 (4)	C19—C18—H18A	116.1
C10—C5—C4	97.9 (3)	C20—C18—H18A	116.1
C15—C5—C4	112.2 (4)	C17—C18—H18A	116.1
C10—C5—C6	107.6 (3)	C20—C19—C18	60.5 (3)
C15—C5—C6	109.8 (4)	C20—C19—H19A	117.7
C4—C5—C6	118.5 (4)	C18—C19—H19A	117.7
O2—C6—C1	106.2 (3)	C20—C19—H19B	117.7
O2—C6—C7	108.4 (3)	C18—C19—H19B	117.7
C1—C6—C7	114.7 (3)	H19A—C19—H19B	114.8
O2—C6—C5	110.8 (3)	C19—C20—C18	60.4 (3)
C1—C6—C5	110.9 (4)	C19—C20—H20A	117.7
C7—C6—C5	105.9 (3)	C18—C20—H20A	117.7
N—C7—C6	106.0 (3)	C19—C20—H20B	117.7
N—C7—C8	116.5 (4)	C18—C20—H20B	117.7
C6—C7—C8	114.1 (3)	H20A—C20—H20B	114.9
N—C7—H7A	106.6	O5—C21—O4	121.4 (5)
C6—C7—H7A	106.6	O5—C21—C22	125.8 (5)
C8—C7—H7A	106.6	O4—C21—C22	112.7 (4)
C9—C8—C7	114.0 (4)	C23—C22—C27	119.4 (5)
C9—C8—H8A	108.8	C23—C22—C21	122.4 (5)
C7—C8—H8A	108.8	C27—C22—C21	118.2 (4)
C9—C8—H8B	108.8	C22—C23—C24	119.9 (5)
C7—C8—H8B	108.8	C22—C23—H23A	120.1
H8A—C8—H8B	107.7	C24—C23—H23A	120.1
C10—C9—C14	116.2 (4)	C23—C24—C25	120.2 (5)
C10—C9—C8	118.0 (4)	C23—C24—H24A	119.9
C14—C9—C8	125.8 (4)	C25—C24—H24A	119.9
C11—C10—C9	123.0 (4)	C26—C25—C24	119.1 (6)
C11—C10—C5	109.3 (4)	C26—C25—H25A	120.4
C9—C10—C5	127.6 (4)	C24—C25—H25A	120.4
C10—C11—O3	112.5 (4)	C25—C26—C27	120.8 (6)
C10—C11—C12	120.5 (4)	C25—C26—H26A	119.6
O3—C11—C12	126.9 (4)	C27—C26—H26A	119.6
C11—C12—C13	117.8 (4)	C26—C27—C22	120.4 (5)
C11—C12—O4	122.4 (4)	C26—C27—H27B	119.8
C13—C12—O4	119.5 (4)	C22—C27—H27B	119.8
			
C6—C1—C2—C3	59.3 (6)	C6—C5—C10—C11	146.1 (4)
C1—C2—C3—O1	135.8 (5)	C15—C5—C10—C9	87.6 (5)
C1—C2—C3—C4	−44.0 (6)	C4—C5—C10—C9	−155.2 (5)
C11—O3—C4—C5	29.6 (5)	C6—C5—C10—C9	−31.9 (6)
C11—O3—C4—C3	−94.4 (4)	C9—C10—C11—O3	172.3 (4)
O1—C3—C4—O3	−34.1 (6)	C5—C10—C11—O3	−5.9 (5)
C2—C3—C4—O3	145.7 (4)	C9—C10—C11—C12	−4.6 (7)
O1—C3—C4—C5	−152.0 (5)	C5—C10—C11—C12	177.3 (4)
C2—C3—C4—C5	27.8 (6)	C4—O3—C11—C10	−15.1 (5)
O3—C4—C5—C10	−31.1 (4)	C4—O3—C11—C12	161.6 (5)
C3—C4—C5—C10	88.0 (4)	C10—C11—C12—C13	2.8 (7)
O3—C4—C5—C15	84.3 (4)	O3—C11—C12—C13	−173.6 (4)
C3—C4—C5—C15	−156.6 (4)	C10—C11—C12—O4	177.0 (4)
O3—C4—C5—C6	−146.1 (4)	O3—C11—C12—O4	0.6 (7)
C3—C4—C5—C6	−27.0 (6)	C21—O4—C12—C11	74.5 (6)
C2—C1—C6—O2	64.0 (5)	C21—O4—C12—C13	−111.5 (5)
C2—C1—C6—C7	−176.4 (4)	C11—C12—C13—C14	0.3 (7)
C2—C1—C6—C5	−56.5 (5)	O4—C12—C13—C14	−174.0 (4)
C10—C5—C6—O2	174.4 (3)	C12—C13—C14—C9	−2.0 (8)
C15—C5—C6—O2	54.8 (5)	C10—C9—C14—C13	0.4 (7)
C4—C5—C6—O2	−75.9 (5)	C8—C9—C14—C13	176.6 (5)
C10—C5—C6—C1	−68.0 (4)	C10—C5—C15—C16	−64.0 (5)
C15—C5—C6—C1	172.4 (4)	C4—C5—C15—C16	−171.9 (4)
C4—C5—C6—C1	41.7 (5)	C6—C5—C15—C16	54.2 (5)
C10—C5—C6—C7	57.1 (4)	C17—N—C16—C15	−172.2 (4)
C15—C5—C6—C7	−62.5 (4)	C7—N—C16—C15	56.8 (5)
C4—C5—C6—C7	166.7 (4)	C5—C15—C16—N	−49.4 (5)
C17—N—C7—C6	166.1 (4)	C7—N—C17—C18	−55.6 (5)
C16—N—C7—C6	−65.2 (4)	C16—N—C17—C18	174.6 (4)
C17—N—C7—C8	−65.8 (5)	N—C17—C18—C19	171.0 (4)
C16—N—C7—C8	62.9 (5)	N—C17—C18—C20	102.6 (5)
O2—C6—C7—N	−52.4 (4)	C17—C18—C19—C20	−110.6 (5)
C1—C6—C7—N	−170.8 (4)	C17—C18—C20—C19	105.6 (5)
C5—C6—C7—N	66.5 (4)	C12—O4—C21—O5	6.6 (7)
O2—C6—C7—C8	178.1 (4)	C12—O4—C21—C22	−175.6 (4)
C1—C6—C7—C8	59.7 (5)	O5—C21—C22—C23	−178.1 (5)
C5—C6—C7—C8	−62.9 (4)	O4—C21—C22—C23	4.3 (6)
N—C7—C8—C9	−88.2 (5)	O5—C21—C22—C27	1.6 (8)
C6—C7—C8—C9	35.8 (5)	O4—C21—C22—C27	−176.1 (4)
C7—C8—C9—C10	−5.0 (6)	C27—C22—C23—C24	0.3 (7)
C7—C8—C9—C14	178.9 (4)	C21—C22—C23—C24	180.0 (4)
C14—C9—C10—C11	2.9 (7)	C22—C23—C24—C25	−1.2 (7)
C8—C9—C10—C11	−173.6 (4)	C23—C24—C25—C26	2.1 (8)
C14—C9—C10—C5	−179.3 (4)	C24—C25—C26—C27	−2.1 (10)
C8—C9—C10—C5	4.2 (7)	C25—C26—C27—C22	1.3 (9)
C15—C5—C10—C11	−94.4 (4)	C23—C22—C27—C26	−0.3 (8)
C4—C5—C10—C11	22.8 (5)	C21—C22—C27—C26	180.0 (5)

### Hydrogen-bond geometry (Å, º)

**Table:**

*D*—H···*A*	*D*—H	H···*A*	*D*···*A*	*D*—H···*A*
O2—H2*A*···N	0.84 (6)	2.18 (7)	2.691 (5)	120 (5)
C24—H24*A*···O5^i^	0.93	2.54	3.453 (7)	168

Symmetry code: (i) *x*+1, *y*, *z*.
